# Effect of maternal positioning during cardiopulmonary resuscitation: a systematic review and meta-analyses

**DOI:** 10.1186/s12884-021-04334-y

**Published:** 2022-02-25

**Authors:** Naosuke Enomoto, Tomoyuki Yamashita, Marie Furuta, Hiroaki Tanaka, Edmond S. W. Ng, Shigetaka Matsunaga, Atsushi Sakurai, on behalf of the Japan Resuscitation Council Maternal task force, Rie Kato, Shinji Takahashi, Jun Takeda, Masahiro Nakao, Eishin Nakamura, Masafumi Nii, Yoshihiro Yamahata, Makoto Tsuji, Takahide Maenaka, Shinji Baba, Yuki Hosokawa, Hiroshi Nonogi, Mayuki Aibiki, Takanari Ikeyama, Tetsuya Isayama, Taku Iwami, Yasuhiro Kuroda, Tetsuya Sakamoto, Naoki Shimizu, Masao Nagayama, Chika Nishiyama, Tetsuo Hatanaka, Shigeharu Hosono, Tasuku Matsuyama

**Affiliations:** 1 Department of Obstetrics and Gynaecology, Graduate School of Medicine, Mie University / Mie University Hospital, 2-174 Edobashi, Tsu, Mie 514-8507 Japan; 2 Department of Emergency and Critical Care Medicine, Japanese Red Cross Medical Center, Tokyo, Japan; 3 Department of Human Health Sciences, Graduate School of Medicine, Kyoto University, Kyoto, Japan; 4 London School of Hygiene & Tropical Medicine, London, UK; 5 Department of Obstetrics and Gynaecology, Saitama Medical Centre, Saitama Medical University, Saitama, Japan; 6 Division of Emergency and Critical Care Medicine, Department of Acute Medicine, Nihon University School of Medicine, Tokyo, Japan

**Keywords:** Maternal cardiac arrest, Cardiopulmonary resuscitation, Pregnant women, Patient positioning

## Abstract

**Background:**

Although rare, cardiac arrest during pregnancy is the leading cause of maternal death. Recently, its incidence has been increasing worldwide because more pregnant women have risk factors. The provision of early, high-quality cardiopulmonary resuscitation (CPR) plays a major role in the increased likelihood of survival; therefore, it is important for clinicians to know how to manage it. Due to the aortocaval compression caused by the gravid uterus, clinical guidelines often emphasise the importance of maternal positioning during CPR, but there has been little evidence regarding which position is most effective.

**Methods:**

We searched the Cochrane Central Register of Controlled Trials, MEDLINE, Embase, and OpenGrey (updated on April 3, 2021). We included clinical trials and observational studies with reported outcomes related to successful resuscitations.

**Results:**

We included eight studies from the 1,490 screened. The eight studies were simulation-based, crossover trials that examine the quality of chest compressions. No data were available about the survival rates of mothers or foetuses/neonates. The meta-analyses showed that resuscitation of pregnant women in the 27°–30° left-lateral tilt position resulted in lower quality chest compressions. The difference is an 19% and 9% reduction in correct compression depth rate and correct hand position rate, respectively, compared with resuscitations in the supine position. Inexperienced clinicians find it difficult to perform chest compressions in the left-lateral tilt position.

**Conclusions:**

Given that manual left uterine displacement allows the patient to remain supine, the resuscitation of women in the supine position using manual left uterine displacement should continue to be supported. Further research is needed to fill knowledge gaps regarding the effects of maternal positioning on clinical outcomes, such as survival rates following maternal cardiac arrest.

## Introduction

Cardiac arrest during pregnancy is rare but life-threatening and involves the lives of two patients: the mother and the fetus [1]. Nationwide population-based studies from the United States, Canada, the United Kingdom and the Netherlands indicate the incidence of maternal cardiac arrest during pregnancy is approximately 1 in 12,000 to 1 in 36,000 [2–5]. The incidence of maternal cardiac arrest and related maternal mortality have increased in several countries over the past 30 years [3, 6, 7]. This increase could be explained partially by more women with risk factors (rising maternal age, obesity and preexisting chronic medical conditions) becoming pregnant [7, 8]. Common causes of maternal cardiac arrest and mortality include anaesthesia complications, bleeding, cardiovascular disease, embolism, uterine atony and hypertension/preeclampsia/eclampsia [6, 9, 10]. Previous studies have suggested that cardiac arrest in pregnant women is more responsive to cardiopulmonary resuscitation (CPR) compared to cardiac arrest in the general population since pregnant women are typically young [11–13].

The rate of maternal survival to hospital discharge for in-hospital maternal cardiac arrest is estimated to be as high as 59% [3, 4], whereas the corresponding figure for maternal cardiac arrest occurring in out-of-hospital settings is much lower, at around 17% [14]. The provision of early, high-quality CPR plays a significant role in increasing the likelihood of survival [15]. Although the resuscitation of a pregnant woman is similar to the standard resuscitation of adults, the physiological changes that occur during pregnancy impose additional clinical challenges [6, 15, 16]. Aortocaval compression occurs beginning around 20 weeks of gestation, when the growth of the uteroplacental unit compresses the aorta, inferior vena cava or both in the supine position [17]. Such compression can reduce cardiac output by as much as 30 to 40% [18]. During CPR, manual chest compressions could produce approximately 30% of the normal cardiac output for the nonpregnant situation [19]. Aortocaval compression in late pregnancy further reduces cardiac output to around 10% of the nonpregnant cardiac output [20, 21].

Clinical guidelines [22–27] recommend relief of aortocaval compression during maternal resuscitation. However, there is no consensus on the best strategy to relieve aortocaval compression during maternal resuscitation. Thus, the latest Royal College of Obstetricians and Gynaecologists guidelines on ‘maternal collapse in pregnancy and the puerperium’ recommended future researchers investigate the effectiveness of CPR with manual uterine displacement versus maternal tilt [20], both of which are considered beneficial in relieving aortocaval compression during chest compressions. A Cochrane systematic review on maternal position during caesarean section for preventing maternal and neonatal complications has been published [17], but the result was based on nonarrest pregnant women, and in light of the quality of chest compression, some strategies that could be effective in relieving aortocaval compression for nonarrest pregnant patients might not be the best option for pregnant women in cardiac arrest.

No current or planned systematic reviews regarding the effects of maternal positioning or strategies were identified in a search of the Cochrane Library, International Prospective Register of Systematic Reviews (PROSPERO) or the Joanna Briggs Institute. Therefore, our systematic review aimed to synthesise the evidence to evaluate the effect of maternal positioning and other strategies during resuscitation to determine which is most effective in improving outcomes following maternal cardiac arrest. Our findings will contribute to evidence-based decision-making for clinicians and provide a basis for the formation of national and international guidelines on the resuscitation of pregnant women.

## Materials and methods

Our review was registered in PROSPERO (CRD42020208177) and conducted in accordance with Preferred Reporting Items for Systematic Reviews and Meta-Analyses (PRISMA) guidelines [28].

### Search strategy

We searched the Cochrane Central Register of Controlled Trials (CENTRAL), MEDLINE, Embase and OpenGrey databases for relevant studies on 16 November 2019, and we updated them on 3 April 2021. We did not restrict the publication year. We also checked the reference lists of all included studies and relevant existing systematic reviews for additional studies. We used subject headings in combination with key words. We devised three sets of search terms: (i) population of interest (pregnant women), (ii) health condition of interest (cardiac arrest) and (iii) intervention (or exposure) evaluated (Table 1).

**Table 1:** Search strategy (Medline OvidSP) 1946 to April 2021

1	exp Pregnancy Complications, Cardiovascular/ or exp Pregnancy/ or exp Pregnancy, High-Risk/ or exp Pregnancy Complications/
2	exp Pregnant Women/
3	pregnan*.mp.
4	matern*.mp.
5	exp Maternal Mortality/ or exp Maternal Death/
6	(maternal adj3 morbidit*).mp.
7	exp Obstetrics/
8	obstetric*.mp.
9	Pregnant wom#n.mp.
10	parturient.mp. or exp Labor, Obstetric/ or exp Anesthesia, Obstetrical/
11	peripartum.mp. or exp Peripartum Period/
12	exp Perinatology/
13	Perinatal.mp.
14	gestation*.mp.
15	gravid*.mp.
16	matern*.mp.
17	1 or 2 or 3 or 4 or 5 or 6 or 7 or 8 or 9 or 10 or 11 or 12 or 13 or 14 or 15 or 16
18	exp Heart Arrest/
19	(heart adj5 arrest?).mp.
20	(cardiac adj5 arrest?).mp.
21	(cardiopulmonary adj5 arrest?).mp.
22	(cardiovascular adj5 arrest?).mp.
23	asystole?.mp.
24	pulseless electrical activit*.mp.
25	exp Cardiopulmonary Resuscitation/
26	exp Resuscitation/ or exp Out-of-Hospital Cardiac Arrest/
27	CPR.mp.
28	resuscita*.mp.
29	(heart adj3 compression?).mp.
30	(cardiac adj3 compression?).mp.
31	(chest adj3 compression?).mp.
32	(thoracic adj3 compression?).mp.
33	exp Heart Massage/
34	(heart adj3 massage?).mp.
35	(cardiac adj3 massage?).mp.
36	(heart adj3 failure?).mp.
37	(cardiac adj3 failure?).mp.
38	(cardiovascular adj3 failure?).mp.
39	(cardiopulmonary adj3 failure?).mp.
40	(cardiac adj3 collapse?).mp.
41	(cardiovascular adj3 collapse?).mp.
42	(cardiopulmonary adj3 collapse?).mp.
43	cardiovascular.mp. or exp Cardiovascular Diseases/
44	cardiac toxicity.mp. or exp Cardiotoxicity/
45	peri-arrest state?.mp.
46	(life adj3 support*).mp.
47	emergency.mp. or exp Emergencies/ or exp Emergency Medical Services/
48	exp Ventricular Fibrillation/
49	electromechanical dissociation*.mp.
50	AED.mp.
51	18 or 19 or 20 or 21 or 22 or 23 or 24 or 25 or 26 or 27 or 28 or 29 or 30 or 31 or 32 or 33 or 34 or 35 or 36 or 37 or 38 or 39 or 40 or 41 or 42 or 43 or 44 or 45 or 46 or 47 or 48 or 49 or 50
52	uterine displacement.mp.
53	(left adj5 table adj5 tilt).mp.
54	tilt*.mp.
55	(uter* adj5 displac*).mp.
56	left-lateral.mp.
57	(left adj3 lateral).mp.
58	lateral tilt.mp.
59	exp Patient Positioning/
60	Aortocaval compression.mp.
61	(Aort* adj5 compression*).mp.
62	52 or 53 or 54 or 55 or 56 or 57 or 58 or 59 or 60 or 61
63	17 and 51 and 62
64	limit 63 to humans

### Inclusion and exclusion criteria

The study population included pregnant women who experienced cardiac arrest. We made no restrictions regarding maternal age, care settings or nationality. Regarding the intervention, we included studies that examined the effect of maternal positioning or methods to relieve aortocaval compression during CPR. We also included any type of study (randomised control trials [RCTs], nonrandomised clinical trials and observational studies). Because of the rarity of cardiac arrest during pregnancy, we included simulation-based studies using patient mannequins. We excluded reviews and commentaries as well as studies without English language abstracts (Table 2).

**Table 2:** Inclusion and exclusion criteria

	Inclusion criteria	Exclusion criteria
**Population**	• Pregnant women who have experienced cardiac arrest in any settings/countries	• None
**Intervention**	• Any maternal positioning during CPR • Any methods to relieve aortocaval compression during CPR	• None
**Comparators**	• Studies with a comparison (or crossover comparison /any control group) to an intervention group	• Studies with no comparison (control) group
**Outcomes**	• Maternal outcomes: - Return of spontaneous circulation following maternal cardiac arrest - Survival to hospital discharge - Survival with favourable neurologic outcome - Any adverse event • Foetal or neonatal outcomes: - Survival to hospital discharge - Survival with favourable neurologic outcome - Any adverse event • Quality of CPR (e.g. quality of chest compression, quality of ventilation)	• Outcomes with no clinical relevance
	• Experimental studies (RCTs, quasi-RCTs, cross-over trials, etc.) with relevant primary data	• Qualitative studies • Animal studies
**Study design**	• Observational studies (cohort, case-control, cross-sectional studies, etc.) with relevant primary data • Simulation-based studies	
**Language**	• Studies written in English • Studies written in a language other than English that contain an abstract written in English	• Studies without English abstract
**Publication**	• Published and grey literature	• None
**Published year**	• No restriction made	• None

### Outcomes of interest

The primary outcomes of interest included the survival rate of mothers or fetuses/neonates with favourable neurologic outcomes and the return of spontaneous circulation following maternal cardiac arrest. The secondary outcomes of interest were the quality of CPR and any adverse events.

### Study selection

We imported identified studies into Covidence, a web-based tool for systematic reviews. Two review authors (NE and TY) independently screened the studies for relevance based on titles and abstracts; they then screened based on full texts. We resolved any discrepancies via discussions with the review team until we reached a consensus.

### Data extraction and risk of bias assessment

Using data extraction forms designed specifically for this review, two review authors (NE and MF) extracted data from the included studies. We contacted the authors of original studies to obtain missing information and unpublished data. Two review authors (MF and NE) independently assessed the risk of bias in the included studies using a revised Cochrane risk-of-bias tool (RoB2) developed specifically for crossover trials [29] because all studies included in this review applied a crossover design.

### Data synthesis and analysis

Findings from nonrandomised crossover studies are presented narratively. Whenever sufficient data were available from RCTs to estimate the effect size of the intervention, we conducted meta-analyses using Cochrane's Review Manager (RevMan) version 5.3 [30]. We calculated the weighted mean difference and 95% confidence interval (CI) for continuous outcomes. We performed the random effects meta-analyses, because we assumed that the impact of the maternal positioning during cardiopulmonary resuscitation varied from study to study [31]. We assessed clinical heterogeneity (e.g. variability in the interventions such as chest compression on different surfaces; the floor or on the bed) as well as methodological heterogeneity (e.g. variability in study design such as RCTs or nonrandomised studies) within each comparison. Where meta-analyses were performed, we assessed statistical heterogeneity with tau^2^ in addition to visual inspection of the forest plots [32]. We assessed heterogeneity using tau^2^, rather than *I*^*2*^, as tau^2^ is the appropriate measure for indicating the presence of clinically relevant heterogeneity while *I*^*2*^ may be misleading as it depends on the sample size of studies [33]. Furthermore, *I*^*2*^ is not an absolute measure of heterogeneity [34, 35]. We conducted subgroup analyses based on the clinical heterogeneity (chest compression delivery surfaces). If there was a concern to the robustness of the result caused by missing outcome data, sensitivity analysis would have been performed, by comparing results from different methods of dealing with missing data (e.g. available case analysis, imputed case analysis) [36, 37]

### Overall quality of evidence

We used the Grading of Recommendations Assessment, Development and Evaluation (GRADE) approach [38] to assess the body of evidence for all the identified outcomes. We assigned one of four levels — high, moderate, low or very low — to each outcome by considering five domains, including the within-study risk of bias, inconsistency, indirectness, imprecision and publication bias [39]. If sufficient studies had been available (> = 10), then we would have constructed funnel plots to assess publication bias.

## Results

### Search results

**Fig. 1:**
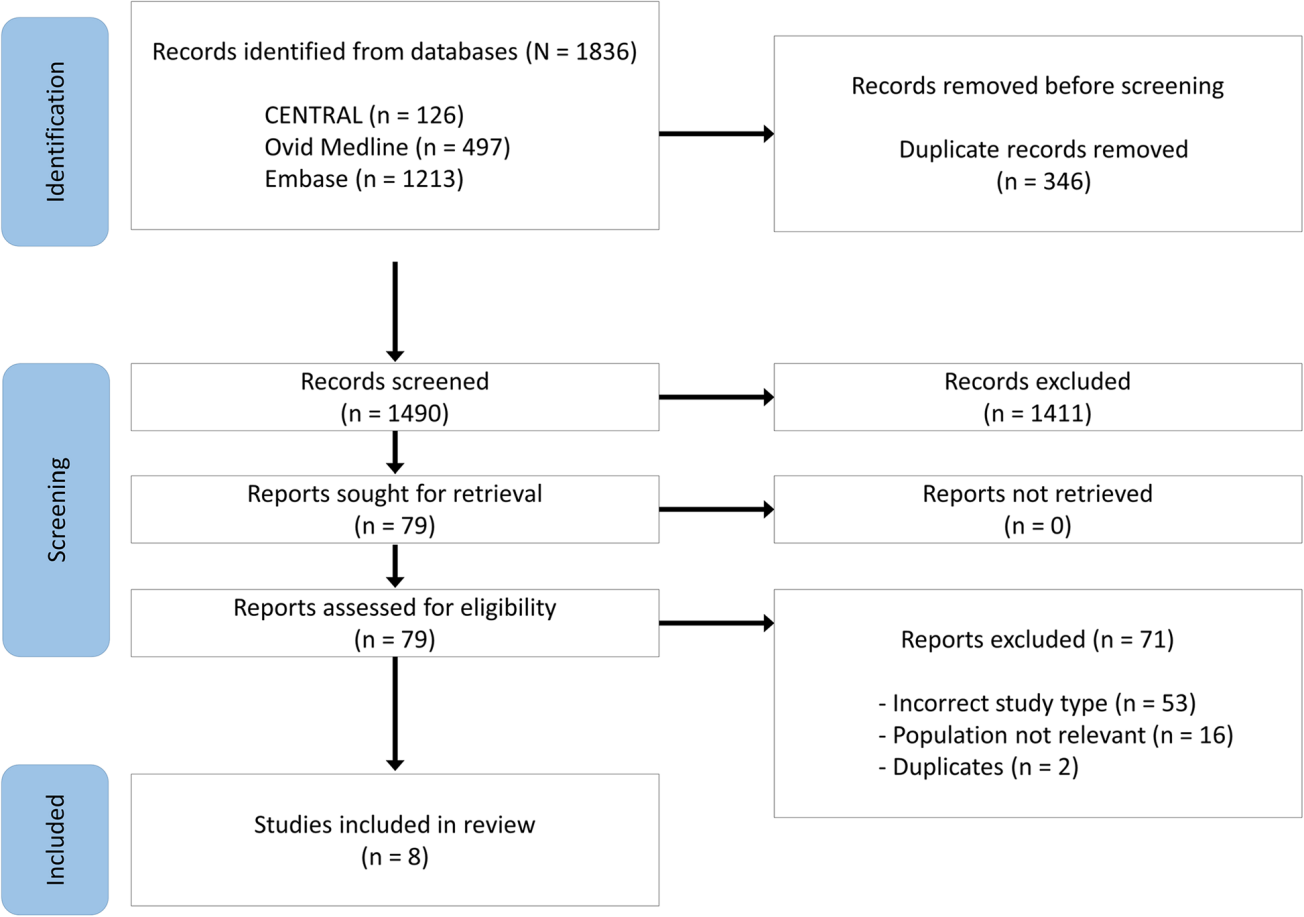
PRISMA flowchart of paper screening process

The databases we searched identified 1,836 articles, including 346 duplicates. We screened a total of 1,490 titles and abstracts and selected 79 articles for full-text evaluation. We identified no additional articles from the reference lists of the included studies or review articles, and of the 79 articles that underwent full-text evaluation, we excluded 71 for the reasons stated in the PRISMA flowchart (Figure 1). A total of eight studies met the inclusion criteria, including six crossover RCTs [40–45] and two nonrandomised crossover studies [46, 47].

### Characteristics of included studies

An overview of included studies is presented in Table 3. All the available studies used mannequins, and none involved living subjects. One crossover RCT [43] examined the effect of manual left uterine displacement in the supine position and compared the results to those in the left-lateral tilt position. Four crossover RCTs [40–42, 44] and one nonrandomised crossover study [46] compared the quality of CPR on a mannequin lying supine (manual left uterine displacement) with that of the left-lateral tilt position. One crossover RCT examined the optimal methods for producing lateral tilt [45], and one nonrandomised crossover study [47] examined the effect of chest compression at various angles between 0° and 90° of inclination. All participants in the included studies were health professionals and performed two or more sequential interventions.

**Table 3:** Characteristics of included studies

Authors/Year	Country	Study Design	Participants	Comparison	Device	Outcomes
Rees and Willis 1988 [47]	UK	Simulation based non-randomised crossover study	8 medical doctors (7 anaesthetists and one cardiologist)	Chest compression (CC) in various angles; 0°, 27°, 32°, 49° and 90° left lateral tilt (LTT)	Calibrated force transducer fitted on the plane	- Resuscitative (compression) force as % of body weight (mean, standard deviation [SD])
Goodwin 1992 [46]	UK	Simulation based non-randomised crossover study	18 midwives	CC in supine without manual left uterine displacement (LUD) vs. CC in lateral tilt produced by human wedge (the degree of tilt not reported)	Laerdal Resusci Anne® Skill Reporting System	- Correct chest compressions (definition not described), % (mean, SD) - Correct expired air ventilations (definition not described), (mean, SD)
Lee et al. 2011 [40]	South Korea	Simulation-based crossover RCT	30 emergency medical residents and technicians	CC in supine without manual LUD vs. CC in 30° LLT surface	Laerdal Resusci Anne® Skill Reporting System (Stavanger, Norway)	- Compression rate, per minute (mean, 95% confidence interval [CI]) - Compression depth, mm (mean, 95% CI) - Correct compression depth rate, 50–60 mm, % (mean, 95% CI) - Correct recoil rate, % (mean, 95% CI) - Correct hand position rate, % (mean, 95% CI) - Highest compression angle (mean, 95% CI) - Lowest compression angle (mean, 95% CI) - Subjective difficulty of CC, 5-point Likert scale (mean, 95% CI)
Kim et al., 2013 [41]	South Korea	Simulation-based crossover RCT	32 BLS-trained medical students (inexperienced rescuers without CPR experience)	CC in supine without manual LUD vs. CC in 30° LLT surface	Laerdal Resusci Anne® Skill Reporting System (Stavanger, Norway)	- Compression rate, per minute (mean, 95% CI) - Compression depth, mm (mean, 95% CI) - Correct compression depth rate, 50–60 mm, % (mean, 95% CI) - Correct recoil rate, % (mean, 95% CI) - Correct hand position rate, % (mean, 95% CI) - Highest compression angle (mean, 95% CI) - Lowest compression angle (mean, 95% CI) - Subjective difficulty of CC, 5-point Likert scale (mean, 95% CI)
Komasawa et al. 2013 [42]	Japan	Simulation-based crossover RCT	27 male medical doctors (with CPR experience)	CC in supine without manual LUD vs. CC in 27° LLT surface (by standing on the left and right sides of the patient)	Laerdal Resuci Anne® Skill Reporting System (Stavanger, Norway)	- Compression rate, per minute (mean, SD) - Compression depth mm (mean, SD) - Correct compression depth rate (> 50 mm), % (mean, 95% CI) - Correct recoil rate, % (mean)
Ip et al. 2013 [45]	UK	Simulation-based crossover RCT	40 healthcare professionals (anaesthetists and midwives)	CC in the LLT with the soft wedge (pillow) vs. firm wedge (foam-rubber) vs. hard wedge (wooden) vs. human wedge	Laerdal Resusci Anne® Skill Reporting System (Kent, UK),	- Compression rate, per minute (mean, 95% CI) - Compression depth mm (median, interquartile range [IQR]) - Correct compression depth rate (> 50 mm), % (median, IQR) - Correct recoil rate (proportion of compressions adequately released), % (mean, 95% CI) - Subjective stability of CC, 5-point Likert scale (median, IQR)
Butcher et al. 2014 [43]	UK	Simulation-based crossover RCT	20 BLS/ALS-trained healthcare professionals (10 anaesthetists and 10 midwives)	CC in supine with manual displacement of uterus vs. CC in LLT produced by a preformed firm-rubber wedge on the floor and on a bed (angle not reported)	Laerdal Resusci Anne® Skill Reporting System (Kent, UK), with ‘pregnancy bump’	- Compression rate, per minute (mean, SD) - Compression depth mm (median, IQR) - Correct compression depth rate (> 50 mm; median, IQR) - Correct recoil rate, % (median, IQR) - Subjective stability and ease of CC, 5-point Likert scale (median, IQR)
Dohi et al., 2017 [44]	Japan	Simulation-based crossover RCT	20 BLS-certified healthcare professionals	CC in supine without manual LUD vs. 30° LLT surface	Laerdal Skill Reporting System	- Compression rate, per minute (mean, SD) - Compression depth mm (mean, SD) - Correct compression depth rate (50–60 mm), % (mean, SD) - Correct recoil rate (within 5 mm of baseline chest height), % (mean, SD) - Correct hand position rate, % (mean, SD) - Subjective ease of CC, 5-point Likert scale (mean, 95%CI)

*Abbreviations*: *RCT* Randomized Controlled Trial, *CC* chest compression, *LUD* left uterine displacement, *LLT* left lateral tilt, *CPR* cardiopulmonary resuscitation, *BLS* Basic life support, *ALS* advanced life support, *SD* standard deviation, *CI* confidence interval, *IQR* interquartile range

### Risk of bias assessment

*Bias due to randomisation*: Of the randomised crossover trials included in this review [40–45], none except one [45] reported the processes used to generate the random allocation sequence and/or allocation concealment. *Bias due to deviations from intended interventions*: Given the nature of the interventions, participants (rescuers) in all studies were aware of their assigned intervention (e.g. chest compression in the supine or lateral tilting positions) during each period of the trial. Four studies [40, 41, 44, 45] ensured a washout period to minimise the carryover effect (after 2 minutes of chest compressions in the first assigned position, the participants rested for 10 minutes to minimise rescuer fatigue), whereas no information was available to assess the carryover effect in the remaining studies [42, 43, 46, 47]. *Bias due to missing outcome data*: There were no missing outcomes [40–44, 46, 47], or the proportion of missing outcomes was small [45]. *Bias due to outcome measurement*: The outcomes were assessed using the PC SkillReporting software system, which was connected to the patient mannequin (Laerdal Resusci Anne®) in all the included studies [40–46] except one [47]. Where the participants could not see the monitor screen displaying the outcomes during the chest compressions, the risk of bias was considered low [41, 44]; however, where information regarding blinding of the outcomes was not provided, the risk of bias was rated as of some concern by taking into account the possibility that knowing the outcomes altered the participants’ performance [40, 42, 43, 45–47]. *Bias due to selection of the reported result*: No studies provided a trial protocol. *Overall*: Six RCTs [40–45] were rated as having some concern for risk bias, whereas the two nonrandomised crossover studies [46, 47] were considered of high risk for bias because at least one domain had a high risk of bias (Table 4).

**Table 4:**
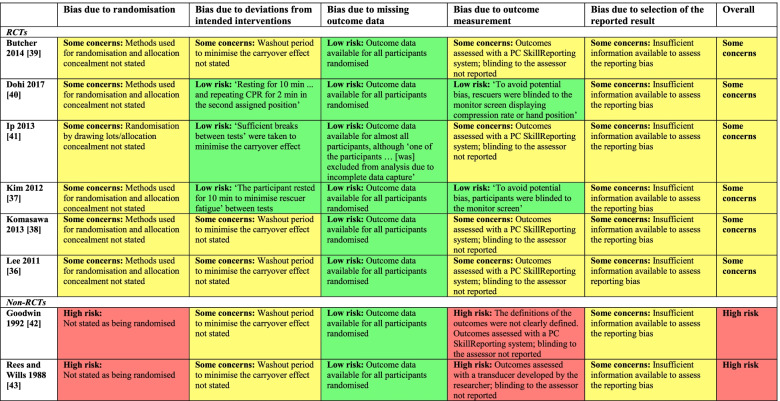
Risk of bias assessment (judgement and supporting evidence) in the included studies using a revised Cochrane risk-of-bias tool for crossover trials

### Intervention effectiveness

#### Maternal and foetal (or neonatal) outcomes

Because all the studies included in this review were conducted on mannequins, no data regarding maternal or foetal/neonatal outcomes were available for our analysis.

#### Quality of CPR and subjective stability/difficulty of chest compression

All eight studies included in this review provided data regarding the quality of the CPR, and some provided data on subjective stability or difficulty (or ease) of chest compression (Table 5).

**Table 5:** Quality of CPR and subjective stability/difficulty of chest compression

**Study**	**Design**	**Comparison groups (Chest compression [CC] from right/left side of patients)**		**Floor/** **Bed**	**N**	**Chest compression rate per minute**			**Correct chest compression depth; %** **(> 50 mm or 50-60 mm)**			**Chest compression depth; mm**			**Recoil rate; %**		
						**Mean (SD or 95%CI)**		***P***	**Mean (SD or 95%CI)**		***P***	**Mean (SD or 95%CI)**		***P***	**Mean (SD or 95%CI)**		***P***
						**Left lateral tilt**	**Supine**		**Left lateral tilt**	**Supine**		**Left lateral tilt**	**Supine**		**Left lateral tilt**	**Supine**	
Comparison 1: Left lateral tilt position (LLT) vs. supine position with manual left uterine displacement																	
Bucher et al. 2014 [43]	RCT	LLT- angle not reported (right)	Supine (right)	Floor	20	115.7 (SD=12.1)	118.5 (SD=12)	NS	median=57 (IQR=17-100)	median=42 (IQR=18-99)	NS^a^	median=43 (IQR=36-50)	median=44 (IQR=36-51)	NS^a^	median=97 (IQR=10-100)	median=80 (IQR=32-100)	NS^a^
				Bed		114.5 (SD=10.0)	116.4 (SD=7.7)	NS	median=25 (IQR=0-89)	median=25 (IQR=7-74)	NS^a^	median=40 (IQR=32-45)	median=40 (IQR=34-46)	NS^a^	median=100 (IQR=71-100)	median=97 (IQR=39-100)	NS^a^
Comparison 2: LLT vs. supine position without manual left uterine displacement																	
Dohi et al. 2017 [44]	RCT	LLT 30° (--)	Supine (--)	Bed	20	123.2 (SD=6.4)	120.5 (SD=6.3)	NS^b^	35.8 (SD=40.1)	76.3 (SD=35.4)	<0.001^b^	--	--	--	100 (SD=0)	100 (SD=0)	NS^b^
Kim et al. 2013 [41]	RCT	LLT 30° (left)	Supine (--)	Floor	32	120.9 (95%CI=115.6-126.1)	121 (95%CI=115.6-126.2)	0.98^c^	64.5 (95%CI=50.5-78.5)	70.2 (95%CI=56.2-84.3)	0.42^c^	52.0 (95%CI=49.2-54.7)	53.3 (95%CI=50.6-56.0)	0.26^c^	99.8 (95%CI=99.3-100.3)	99.4 (95%CI=98.9-99.9)	0.26^c^
Komasawa et al. 2013 [42]	RCT	LLT 27° (left)	Supine (left)	Bed	27	--	--	NS^d^	69.1 (SD=16.0)	85.0 (SD=8.9)	<0.001^d^	49 (SD=3)	52 (SD=2)	0.078^d^	100 (--)	100 (--)	NS^d^
		LLT 27° (right)	Supine (right)	Bed		--	--	NS^d^	27.0 (SD=15.1)	86.1 (SD=8.7)	<0.001^d^	42 (SD=3)	52 (SD=3)	<0.001^d^	100 (--)	100 (--)	NS^d^
Lee et al. 2011 [40]	RCT	LLT 30° (left)	Supine (--)	Floor	30	118.8 (95%CI=114.7-122.9)	121.3 (95%CI=117.2-125.4)	0.07^c^	66.4 (95%CI=55.3-77.4)	87.9 (95%CI=76.8-98.9)	<0.001^c^	52.6 (95%CI=50.4-54.7)	56.1 (95%CI=54.0-58.3)	<0.001^c^	97.3 (95%CI=92.1-102.5)	97.3 (95%CI=92.1-102.5)	0.99^c^
Goodwin 1992 [46]	Non-RCT	probably right lateral tilt	Supine (--)	Floor	18	--	--	--	--	--	--	--	--	--	--	--	--
Comparison 3: Methods for producing LLT (soft wedge vs. firm wedge vs. hard wedge vs. human wedge)																	
Ip et al. 2013 [45]	RCT	LLT with: Soft wedge (pillow) vs. Firm wedge (foam-rubber) vs. Hard wedge (wooden) vs. Human wedge	n/a	Floor	20	Soft: 106.7 (SD=3.8) Firm: 105.6 (SD=3.3) Hard: 105.8 (SD=2.9) human: 106.0 (SD=3.3)	n/a	NS^e^	Overall *soft vs. human* *firm vs. human* *hard vs. human* (lower rate for human wedge; data not reported)	n/a	0.036 *0.047* *0.0033* *0.016*	Overall *soft vs. human* *firm vs. human* *hard vs. human* (lower depth for human wedge; data not reported)	n/a	0.02 *NS* *0.009* *0.015*	Soft: median=99.8 (IQR=96.3-100) Firm: median=99.3 (IQR=93.1-100) Hard: median=99.6 (IQR=95.1-100) Human: median=100 (IQR=98.4-100)	n/a	NS^e^
				Bed	19	Soft: 105.8 (SD=4.3) Firm: 105.4 (SD=2.1) Hard: 104.9 (SD3.6) Human: 106.1 (SD=3.9)	n/a	NS^e^	Overall	n/a	NS	Overall	n/a	NS	Soft: median=99.6 (IQR=94.0-100) Firm: median=100 (IQR=97.7-100) Hard: median=100 (IQR=99.5-100) Human: median=100 (IQR=98.6-100)	n/a	NS^e^
Comparison 4: Various angles (0°, 27°, 32°, 49° and 90°) of inclination																	
Rees and Willis 1988 [47]	Non-RCT	LLT 27°, 32°, 49°, and 90° (left)	Supine (--)	Floor	7	--	--	--	--	--	--	--	--	--	--	--	--
**Study**	**Design**	**Comparison groups (Chest compression [CC] from right/left side of patients)**		**Floor/** **Bed**	**N**	**Correct hand position rate; %**			**Resuscitation (compression) force; %**			**Correct chest compressions; %**			**Subjective stability of CC**		
						**Mean (SD or 95%CI)**		***p***	**Mean (SD or 95%CI)**		***p***	**Mean (SD or 95%CI)**		***p***	**Median (IQR)**		***p***
						**Left lateral tilt**	**Supine**		**Left lateral tilt**	**Supine**		**Left lateral tilt**	**Supine**		**Left lateral tilt**	**Supine**	
Comparison 1: Left lateral tilt position (LLT) vs. supine position with manual left uterine displacement (LUD)																	
Bucher et al. 2014 [43]	RCT	LLT- angle not reported (right)	Supine (right)	Floor	20	--	--	--	--	--	--	--	--	--	median=4.0* (IQR=3-4)	median=4.5* (IQR=4-5)	0.048^a^
				Bed		--	--	--	--	--	--	--	--	--	median=3.0* (IQR=3-3)	median=4.0* (IQR=4-4)	0.007^a^
Comparison 2: LLT vs. supine position without manual left uterine displacement																	
Dohi et al. 2017 [44]	RCT	LLT 30° (--)	Supine (--)	Bed	20	88.8 (SD=28.6)	99.7 (SD=1.1)	<0.05^b^	--	--	--	--	--	--	--	--	--
Kim et al. 2013 [41]	RCT	LLT 30° (left)	Supine (--)	Floor	32	72.0 (95%CI=59.8-84.2)	78.1 (95%CI=65.8-90.3)	0.47 ^c^	--	--	--	--	--	--	--	--	--
Komasawa et al. 2013 [42]	RCT	LLT 27° (left)	Supine (left)	Bed	27	--	--	--	--	--	--	--	--	--	--	--	--
		LLT 27° (right)	Supine (right)	Bed		--	--	--	--	--	--	--	--	--	--	--	--
Lee et al. 2011 [40]	RCT	LLT 30° (left)	Supine (--)	Floor	30	75.8 (95%CI=63.0-88.6)	84.9 (95%CI=72.2-97.7)	0.09^c^	--	--	--	--	--	--	--	--	--
Goodwin 1992 [46]	Non-RCT	probably right lateral tilt	Supine (--)	Floor	18	--	--	--	--	--	--	67.6 (SD=21)	32.5 (SD=24.9)	0.0005^f^	--	--	--
Comparison 3: Methods for producing LLT (soft wedge vs. firm wedge vs. hard wedge vs. human wedge)																	
Ip et al. 2013 [45]	RCT	LLT with: Soft wedge (pillow) vs. Firm wedge (foam-rubber) vs. Hard wedge (wooden) vs. Human wedge	n/a	Floor	20	--	--	--	--	--	--	--	--	--	Soft: median=3* (IQR=2-4) Firm: median=4* (IQR=3-4) Hard: median=5* (IQR=5-5) Human: median=3* (IQR=1-4)	--	<0.0001
				Bed	19	--	--	--	--	--	--	--	--	--	Soft: median=2* (IQR=1-3) Firm: median=4* (IQR=3-4) Hard: median=4* (IQR=3-4) Human: median=4* (IQR=3-5)	--	<0.0001
Comparison 4: Various angles (0°, 27°, 32°, 49° and 90°) of inclination																	
Rees and Willis 1988 [47]	Non-RCT	LLT 27°, 32°, 49°, and 90° (left)	Supine (--)	Floor	7	--	--	--	LLT 27°: 55.3 (SD=5.5) LLT 32°: 46.4 (SD=3.9) LLT 49°: 41.5 (SD=3.5) LLT 90°: 36.3 (SD=5.4)	66.7 (SD=6.5)	--	--	--	--	--	--	--
**Study**	**Design**	**Comparison groups (Chest compression [CC] from right/left side of patients)**		**Floor/Bed**	**N**	**Subjective difficulty of CC**			**Subjective ease of CC**			**Correct expired air ventilations**					
						Mean (SD or 95%CI)		*P*	Mean (SD or 95%CI)		*P*	Mean (SD or 95%CI)		*P*			
						Left lateral tilt	Supine		Left lateral tilt	Supine		Left lateral tilt	Supine				
Comparison 1: Left lateral tilt position (LLT) vs. supine position with manual left uterine displacement (LUD)																	
Bucher et al. 2014 [43]	RCT	LLT- angle not reported (right)	Supine (right)	Floor	20	--	--	--	median=3.0* (IQR=3-4)	median=4.0* (IQR=4-4)*	0.011^a^	--	--	--			
				Bed		--	--	--	median=4.0* (IQR=3-4)	median=5.0 (IQR=4-5)	NS	--	--	--			
Comparison 2: LLT vs. supine position without manual left uterine displacement																	
Dohi et al. 2017 [44]	RCT	LLT 30° (--)	Supine (--)	Bed	20	--	--	--	3.95^†^ (95%CI=3.68-4.22)	1.75^†^ (95%CI=1.31-2.19)	<0.001^b^	--	--	--			
Kim et al. 2013 [41]	RCT	LLT 30° (left)	Supine (--)	Floor	32	68.8^**‡**^ (95%CI=62.8-74.9)	58.3^**‡**^ (95%CI=52.2-64.4)	0.007^c^	--	--	--	--	--	--			
Komasawa et al. 2013 [42]	RCT	LLT 27° (left)	Supine (left)	Bed	27	--	--	--	--	--	--	--	--	--			
		LLT 27° (right)	Supine (right)	Bed		--	--	--	--	--	--	--	--	--			
Lee et al. 2011 [40]	RCT	LLT 30° (left)	Supine (--)	Floor	30	68.4^**‡**^ (95%CI=62.1-74.8)	64.4^**‡**^ (95%CI=58.2-71.0)	0.28^c^	--	--	--	--	--	--			
Goodwin 1992 [46]	Non-RCT	probably right lateral tilt	Supine (--)	Floor	18	--	--	--	--	--	--	56.7 (SD=27.7)	62.2 (SD=21.4)	NS			
Comparison 3: Methods for producing LLT (soft wedge vs. firm wedge vs. hard wedge vs. human wedge)																	
Ip et al. 2013 [45]	RCT	LLT with: Soft wedge (pillow) vs. Firm wedge (foam-rubber) vs. Hard wedge (wooden) vs. Human wedge	n/a	Floor	20	--	--	--	--	--	--	--	--	--			
				Bed	19	--	--	--	--	--	--	--	--	--			
Comparison 4: Various angles (0°, 27°, 32°, 49° and 90°) of inclination																	
Rees and Willis 1988 [47]	Non-RCT	LLT 27°, 32°, 49°, and 90° (left)	Supine (--)	Floor	7	--	--	--	--	--	--	--	--	--			

*Note*: *CC* chest compression, *LUD* left, *LLT* left lateral tilt, *NS* not significant

For Dohi et al. 2017, data were obtained from the authors

Statistical analysis used in the original studies: a) Wilcoxon signed rank sum; b) Student’s t-test; c) mixed model; d) two-way repeated ANOVA; d) repeated ANOVA; f) paired t-tests; unless specified, statistical tests used were not reported in the original studies

* 5-point Likert-scale: 1=extremely (or very) poor; 2=poor; 3=adequate; 4=good; 5=excellent or very good (higher score =better stability or easier)

† 5-point Likert-scale: 1=very easy; 2=easy; 3=normal; 4=difficult; 5=very difficult (higher score = more difficult)

**‡** Visual analogue scale from 0 mm=extremely east to 100 mm=extremely difficult (higher score = more difficult)

##### Comparison 1: Left lateral tilt position vs. manual left uterine displacement

*Quality of chest compression*

Based on one crossover RCT [43] involving 20 health professionals, there was no statistically significant difference in the quality of chest compressions as measured with compression rates, compression depth, correct compression depth (> 50 mm) rates and correct recoil rates between the manual left uterine displacement in the supine position and the left lateral tilt position produced by a firm-rubber wedge. The results were consistent both on the floor and on the bed. The mean compression rates observed ranged from 114.5/min to 118.5/min and were within the range of adequate compression rates recommended by clinical guidelines. However, insufficient compression depth (median ranging from 40 to 44 cm) and low rates of correct compression depth (median ranging from 25% to 57%) were observed across all groups, indicating generally poor performance of chest compressions in the sample of this study.

*Subjective ease and stability of chest compression*

One study [43] involving inexperienced rescuers reported greater ease and stability of chest compressions in the supine position with manual left uterine displacement than in the left-lateral tilt position; the differences were statistically significant.

##### Comparison 2: left lateral tilt position (27°–30°) vs. supine position without manual left uterine displacement

*Quality of chest compression*

A total of five studies including four crossover RCTs [40–42, 44] and one nonrandomised crossover study [46] provided data on this outcome. Due to the methodological heterogeneity (i.e. RCTs or nonrandomised studies), only RCTs were included in the meta-analyses and results from nonrandomised study were presented separately in narrative form.

**Fig. 2:**
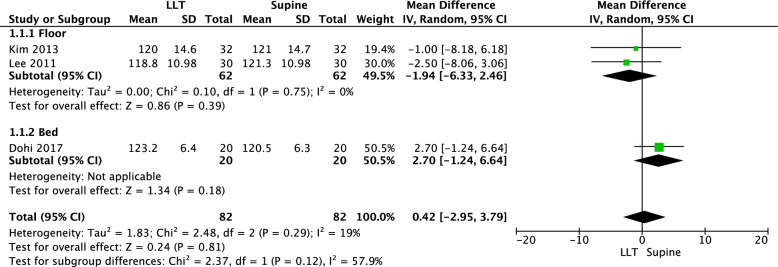
Meta-analysis of the mean difference in chest compressions per minute

The four RCTs consistently showed no statistically significant differences between the supine and the left-lateral tilt position groups. Of these RCTs, one [42] was excluded from the meta-analysis (Fig. 2) because of insufficient data provided in the original study (the means and standard deviations were unreported).

**Fig. 3:**
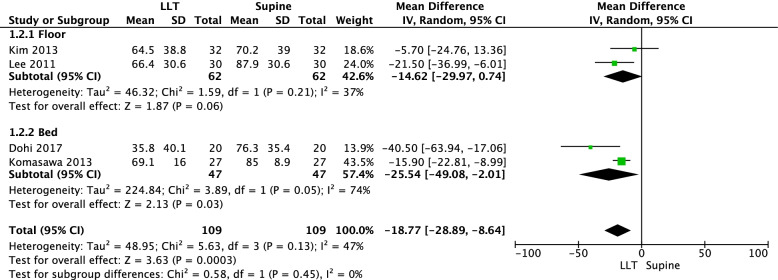
Meta-analysis of the mean difference in the rate of correct chest compression depth (%)

A total of four RCTs [40–42, 44] assessed the rate of correct chest compression depth. The meta-analysis of these RCTs revealed the mean percentage of correct chest compression depth decreased by 18.77% when the left-lateral tilt position was used instead of the supine position; the difference was statistically significant (four RCTs, mean difference [MD] = -18.77, 95% CI = -28.89, -8.64, tau^2^ =48.95, I^2^ = 47%; Fig. 3). Subgroup analyses stratified by chest compression delivery surfaces (floor or bed) resulted in similar findings.

**Fig. 4:**
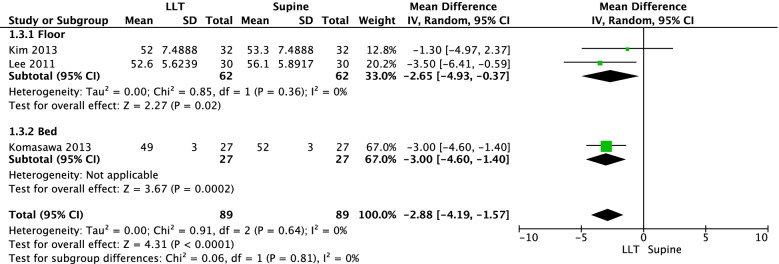
Meta-analysis of the mean difference in the chest compression depth (mm)

In addition, a meta-analysis of three RCTs [40–42] including a total of 89 health professionals revealed the mean chest compression depth was 2.88 mm lower in the 27°–30° left-lateral tilt position than in the supine position; the difference was statistically significant (three RCTs, MD = -2.88 mm, 95% CI = -4.19, -1.57, tau^2^ = 0, I^2^ = 0%; Fig. 4). The results were consistent across subgroups defined by the surface (floor or bed).

A total of four RCTs [40–42, 44] reported the recoil rates, none of which indicated statistically significant differences between the supine and the left-lateral tilt groups, either on the floor or on the bed.

**Fig. 5:**
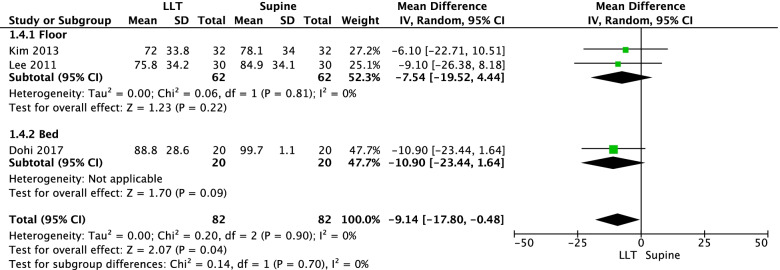
Meta-analysis of the mean difference in the correct hand position rate (%)

A total of three RCTs [40, 41, 44] reported the rate of correct hand positioning during chest compressions. The results of the meta-analysis indicated the correct hand position rate was 9% lower with the patient mannequin in the left-lateral tilt position than with it in the supine position (three RCTs, MD = -9.14, 95% CI = -17.8, -0.48, tau^2^ = 0, I^2^ = 0%; Figure 5).

There was one non-randomised crossover study conducted in 1992 [46] which reported that chest compression was significantly better (with the mean percentage of correct cardiac compression being approximately 34% higher) in the wedged position than in the supine position. The author stated that a common reason for inaccuracies is ‘compression of too great a force’ ([46], p. 434), but neither the definition of correct cardiac compression nor compression force was provided.

*Subjective difficulty (or ease) of chest compressions*

Two RCTs [41, 44] involving both experienced and inexperienced rescuers reported that performing chest compressions in the left-lateral tilt position was significantly more difficult than doing so in the supine position, whereas another RCT [40] including only experienced emergency medical doctors reported no difference in the subjective difficulty between the two positions.

*Quality of ventilation*

One nonrandomised crossover study [46] involving 18 midwives reported there was no statistically significate difference in the percentage of correct expired air ventilations (during performance of mouth-to-mouth resuscitation) between the supine and the left literal tilt positions (mean [SD] = 62.2% [21.4] in the supine vs. 56.7% [27.7] in the left literal tilt positions). However, the definition of correct expired air ventilations was not described in the original study; it noted only that the commonest course of inaccurate ventilation was the ventilation of small volume.

##### Comparison 3: methods for producing left lateral tilt position (soft vs. firm vs. hard vs. human wedge)

*Quality of chest compression*

One crossover RCT reported that the type of wedge — the soft wedge (pillow), firm wedge (foam-rubber), hard wedge (wooden) or human wedge — had no effect on the average rate or adequate release of chest compressions. The study consistently indicated that the depth of compressions (compression depth [mm] and rate of correct compression depth > 50 mm) was reduced with the human wedge compared with other wedges; the differences were statistically significant during chest compressions on the floor but not on the bed.

*Subjective stability of chest compressions*

One crossover RCT reported that the firm and hard wedges were the most stable (stability rated as ‘good’ or ‘very good’), whereas the soft wedges were the least stable during chest compressions during chest compression on either the floor or bed.

##### Comparison 4: chest compressions in various angles (0°, 27°, 32°, 49° and 90°) of inclination

*Quality of chest compression*

In one nonrandomised trial [47] involving eight medical doctors, the maximum possible resuscitative force (as measured with calibrated force transducer fitted on the plane) decreased as the angle of inclination of the plane increased, from 67% of body weight in the supine position to 36% in the full lateral.

## Discussion

Our systematic review evaluated the effect of maternal positioning for successful resuscitation of pregnant women. We identified no studies that evaluated the outcomes with real maternal patients. However, there were eight simulation-based crossover trials (six RCTs and two non-RCTs) that specifically examined the impact of maternal positioning or strategies on the quality of chest compression for hypothetical cardiac arrest maternal patients using a mannequin. Overall, meta-analyses of RCTs indicated resuscitation in the supine position enhances the quality of chest compressions by increasing the rates of correct compression depth and correct hand position, compared with resuscitation in the 27°–30° left-lateral tilt position in pregnant women. The results were consistent for chest compressions performed both on the bed and on the floor. This review also suggested chest compressions in the left-lateral tilt position may be more difficult than chest compressions in the supine position for inexperienced health professionals.

### Quality of evidence

For all the outcomes included in this review, the quality of evidence was rated as very low using the GRADE criteria. The certainty of evidence was downgraded for risk of bias, indirectness, inconsistency and imprecision of results. More specifically, we downgraded one level for a potential risk of bias due to the randomisation process, insufficient washout period between phases and/or unblinding of participants for all the outcomes measuring for quality of CPR. We also downgraded two levels for indirectness of evidence because all the outcome was assessed with simulation-based studies using hypothetical cardiac arrest maternal patient mannequins. We further downgraded one level for serious unexplained inconsistency (heterogeneity) for quality of chest compression as measured with correct chest compression depth rate and another one for serious imprecision (wide confidence intervals) of the mean effect for correct chest compression depth rate and correct hand position rate.

Study effect estimates for quality of chest compression varied between RCTs and nonrandomised study with conflicting results; RCTs favouring the spine position and nonrandomised study [46] favouring the left-lateral tilt position. The nonrandomised study was published in 1992, whereas RCTs were published more recently, in the 2010s.Some of this variation is likely to be caused by differences in the definition used for measuring quality of chest compression that reflect changes in the clinical guidelines’ recommendations for CPR in past decades, which we discuss further in the Comparison with Existing Guidelines and Reviews section below. There is a lack of clarity of the definition of high-quality chest compression and high risk of selection bias due to a lack of randomisation in the non-randomised study. Therefore, we only included the results from RCTs in the meta-analyses.

Heterogeneity is not a reason for downgrading the evidence for quality of CPR apart from quality of chest compression as measured using the percentage of correct chest compression depth (Tau^2^ = 48.95, I^2^ = 47%). The percentage of correct chest compression depth was consistently lower in the left-lateral tilt position than the supine position. However, the effect of size (mean difference in the percentage of correct chest compression depth) varied from study to study: three appear to have large effects (15.9–40.5%) and one much smaller effect (5.7%). There are many probable causes of heterogeneity, which cannot be explained by a subgroup analysis by chest compression delivery surfaces (floor or bed) or study population (experienced or inexperienced rescuers). The estimated effect of maternal positioning is larger, 57% [42], when a chest compression was performed from patients’ right side, showing lower correct percentage (27%) in the left-lateral tilt position, compared with the spine position (86%). We need more studies to gain a reliable estimate of heterogeneity and reasons for it.

### Comparison with existing guidelines and reviews

The 2020 American Heart Association (AHA) Guidelines [48] recommend that “priorities for the pregnant woman in cardiac arrest should include provision of high-quality CPR and relief of aortocaval compression through left-lateral uterine displacement” (Supplement, p. 454). This recommendation is based primarily on the physiology of pregnancy, extrapolations from the non-arrest pregnancy states [49, 50] and non-randomised simulation-based studies [46, 47]. However, the interpretation of the recommendation is not straightforward because the recommendation was based on inconsistent results, including the non-randomised simulation-based studies [46, 47] conducted 30 to 40 years ago.

One of the studies often utilised in clinical practice guidelines is Goodwin’s non-randomised simulation-based study published in 1992 [46]. Goodwin found that chest compression quality was more reduced in the supine position than in the wedged position, using the human wedge manoeuvre. According to Goodwin, the common reason for inaccuracy is “compression of too great a force” ([46], p. 434), but the correct definition of cardiac compression and compression were not provided. Our systematic review revealed that findings from recent RCTs contradict Goodwin’s findings, possibly because of changes in CPR recommendations in past decades [51]. For example, the current CPR guidelines recommend a target chest compression depth of 5–6 cm, whereas it was 4–5 cm (AHA Guidelines 2005) in the past. Even further back, it was defined as the difference in the height of a rescuer’s shoulder, not in a victim’s chest, using 2.5–5 cm in the 1992 AHA Guidelines [52].

Although our review included only indirect evidence from simulation-based studies, the trials included had more sophisticated studies that overcome the methodological limitations commonly observed in previous studies (such as lack of randomisation, the potential risk of carryover effect and the inaccuracy of measuring outcomes). Our results showed that resuscitation in the supine position enhances the quality of the resuscitation activity. Together with evidence from previous systematic reviews on the non-arrest pregnant population [17, 53] that shows that manual left uterine displacement effectively relieves aortocaval pressure in pregnant women with hypotension, it is reasonable to conclude that manual left uterine displacement in the supine position is more effective than a left-lateral tilt position to increase the chest compression quality during resuscitation. This can, in turn, contribute to increased maternal and foetal survival rates following maternal cardiac arrest.

### Strengths and limitations of the review

Given its systematic and comprehensive literature search, our review enhanced evidence regarding the effect of maternal positioning during maternal CPR, particularly on the quality of chest compressions. Where we found information in the included studies to be insufficient, we contacted the original researcher, if doing so was possible. However, the quality of evidence produced by our systematic review was still poor, mainly because of indirect evidence from the mannequin studies.

We did not identify any study that evaluated the effect of maternal positioning using real patients. Therefore, there were no data on survival rates and the return of spontaneous circulation following maternal cardiac arrest. Foetal/neonatal outcomes were also unavailable. Thus, the only outcomes available constituted indirect evidence of the quality of CPR, which was obtained from simulation-based studies using hypothetical cardiac arrest maternal patient mannequins. Therefore, there are serious limitations regarding the applicability and transferability of the findings of our systematic review to real maternal patients.

From the study design point of view, all studies included in our review were crossover trials in which each healthcare professional involved performed chest compressions on a mannequin in two or more maternal positions in random order. Because each participant acted as their own control, this design allowed them to express the difficulty with chest compressions that they experienced during a particular maternal position. The crossover trials could have provided more precise effect size estimates than parallel-group trials if appropriate statistical analyses (paired analyses) had been applied [54, 55]. However, this was not the case in some of our included studies. Data on within-subjects correlation were unavailable, so this advantage of a crossover design could not be utilised. Our meta-analysis estimated the average effect of an intervention (maternal positioning), but given the small number of studies to be synthesised for each outcome, the statistical model used for the meta-analysis (random effects model) could not *estimate* the between-study variance (the extent of variation among the effects observed in different studies).

### Further research

Knowledge gaps still exist concerning the effect and efficacy of CPR with manual uterine displacement versus tilt positioning on clinical outcomes following maternal cardiac arrest. Simulation-based RCTs specifically designed to evaluate the favourable or unfavourable effects of manual left uterine displacement should be carried out to assess the quality of CPR, including delay and interruption of CPR in relation to performing manual left uterine displacement. Further studies also must focus on establishing what could be the best strategies (including for manual left uterine displacement) for high-quality CPR. This is important because there are various recommendations regarding manual left uterine displacement, possibly referencing the situations or settings wherein maternal cardiac arrest occurs. For example, guidelines recommend ‘placing a hand below the uterus on the maternal right and pushing the uterus slightly upwards and to the left’ ([50], p.29), which can be done with one-hand or two-hand techniques and from the left or right side of the patient [24, 48, 56]. There is, however, a lack of evidence about whether and how these different strategies affect the quality of CPR. Because maternal cardiac arrest is rare and RCTs evaluating the effects of maternal position with real patients would be unrealistic, the development of a nationwide database that collects data concerning both in-hospital and out-of-hospital maternal cardiac arrest patients would be beneficial. Such a database would be critical to predict the clinical outcomes of such cases, including the survival rates of mothers and babies with favourable neurologic outcomes, after cardiac arrest vis-à-vis the strategies used for relieving aortocaval compression during maternal resuscitation.

## Conclusion

Although rare, cardiac arrest during pregnancy is the leading cause of maternal death. Recently, its incidence has been increasing worldwide because more pregnant women have risk factors. The provision of early, high-quality cardiopulmonary resuscitation (CPR) plays a major role in the increased likelihood of survival. Therefore, clinicians should be familiar with its management. Because of the aortocaval compression caused by the gravid uterus, clinical guidelines often emphasise the importance of maternal positioning during CPR, but there has been little evidence regarding which position is most effective. Our systematic review synthesised evidence from trials published in recent years, which should provide guidance on updating clinical practice guidelines. The meta-analyses showed that resuscitation of pregnant women in the 27°–30° left-lateral tilt position resulted in lower quality chest compressions. The difference is an 19 and 9% reduction in compression depth rates and hand position, respectively, than resuscitations in the supine position. Inexperienced clinicians find it difficult to perform chest compressions in the left-lateral tilt position. Given that manual left uterine displacement allows the patient to remain supine, the resuscitation of women in the supine position using manual left uterine displacement should continue to be supported. Further research is needed to fill knowledge gaps regarding the effects of maternal positioning on clinical outcomes, such as survival rates following maternal cardiac arrest.

## Data Availability

The datasets used and/or analysed during the current study are available from the corresponding author on reasonable request.

## Abbreviations

ALS: Advanced life support
BLS: Basic life support
CPR: Cardiopulmonary resuscitation
LLT: Left lateral tilt
LUD: Left uterine displacement
RCT: Randomised Controlled Trial
